# Sustainable return to work after depression - A comparative study among occupational physicians and affected employees

**DOI:** 10.3389/fpubh.2022.946396

**Published:** 2022-10-06

**Authors:** Richard Paulinus Ersel, Roman Pauli, Petra Maria Gaum, Jessica Lang

**Affiliations:** Institute for Occupational, Social and Environmental Medicine, RWTH Aachen University, Aachen, Germany

**Keywords:** depression, return to work, sick leave, workplace, occupational health physicians

## Abstract

**Introduction:**

The number of sick days taken from work due to depression is steadily rising. A successful return to work (RTW) is essential for sustainable reintegration. This study aims to identify factors to optimize RTW and to investigate approaches for sustainable RTW (sRTW) after depressive episodes.

**Methods:**

Semi-structured expert interviews with senior occupational physicians (OPs, *N* = 5) served to develop two surveys among OPs (*N* = 180) and employees after depressive episode (*N* = 192). Predictors of RTW rating, workplace-based RTW interventions and sRTW interventions were analyzed using multiple hierarchical regression, chi-square difference and *t*-tests.

**Results:**

For OPs, employee training on mental illness prevention was found to be the strongest predictor of overall RTW rating, whereas understanding and appreciation in conversations and stigmatization were strongest predictors of overall RTW rating by the employees. Compared to the employees, OPs reported significantly more availability of workplace-based interventions. To prevent relapse, the employees prioritized sufficient time and financial security during the RTW process more than OPs.

**Conclusions:**

The study identified facilitating and hindering factors that can inform further research and practice to improve RTW after depressive episodes. To redress the awareness gap about the availability of workplace-based interventions, regular contact between OPs and employees is crucial. Several factors were considered to be of varying importance for relapse prevention by the two groups. Multiple perceptions and needs ought to be taken into account during RTW.

## Introduction

Among the 20 leading causes of global years lived with disability (YLD), major depression ranks second after back pain (1). The United States documented an increase in the prevalence of depressive symptomatology among adults from 20.9% in 2005 to 25.7% in 2010 (2). Globally, the surge in the prevalence of major depressive disorder has been exacerbated by the impact of the COVID-19 pandemic (3). Similarly, the number of days of sick leave due to depressive episodes has been growing steadily in Germany (4) as the majority of people affected are of working age (5). Compared to those affected by physical illnesses, employees with depression are absent from work for longer periods (6). Depressive symptoms are strong predictors of long-term work disability and absenteeism (7). In this study, we investigated the indicators of successful return to work (RTW) and sustainable RTW (sRTW) after depressive episodes. We defined RTW as successful when the employee has fully returned to work after sickness absence due to depression. This also means that the employee works the same hours as before the sickness absence. Currently, there is no standard definition of sRTW. In the literature, periods of 30 days (8) or 3 months (9) without depression relapse after the day of successful RTW are defined as sRTW, keeping in mind that the risk of early relapse is quite high (10). Therefore, in this study, we used the prevention of depression relapse from the date of successful RTW to operationalize sRTW.

The etiology of depression is complex and includes both non-modifiable (e.g., age or gender) and modifiable, as well as non-work-related and work-related, risk factors (11). This study wants to take over an applied occupational health perspective and thus focusses on the modifiable work-related risk factors, which are stressors such as high work demands, low job control or lacking support from coworkers or supervisors, because they predict stress-related disorders (12). Stress-related disorders include depression, which can lead to the loss of pleasure in almost all activities (13), reduced work performance and sickness absence from work (7). The inability to work for an extended period of time, combined with limitations due to the mental disease, can have a major impact on employees' quality of life (14) as well as the healthcare system economy (15). Because of the negative outcomes for the healthcare system and employees, a successful and sustainable RTW is of crucial importance. Occupational physicians (OPs) are key stakeholders in the RTW process.

OPs' responsibilities include maintaining employees' ability to work, preventing occupational diseases, providing preventive health care, and supporting occupational rehabilitation and reintegration (16). In general, OPs should have a good insight into the employees' work environments (17) and play a pivotal role in supporting the RTW process by suggesting therapeutic and organizational interventions for RTW and helping the employees apply the interventions in the workplace (18). Based on individual circumstances, illness characteristics and specific work environment, the OP and the employee may decide together which interventions can optimize the RTW process.

In our study, the employees reported the feeling of being taken seriously and being understood by the supervisors as beneficial when returning to work after a depression (19). Recent research has sought to examine the role of the supervisors during RTW. While Negrini et al. (20) assume that the supervisors' attitudes and behaviors have an important impact on the RTW of employees with depression, Ervasti et al. (21) have not found better supervisor-employee communication to be associated with quicker RTW. However, a work environment that is sensitive to mental health issues can facilitate RTW (22), whereas prejudices and stigmatization due to mental illness, or an unhealthy work climate, can complicate or even hinder the RTW process (22). The lack of collegial support can impede interventions during RTW (21) given that work relationships have an elevated importance to individuals suffering from mental illnesses compared to those with physical disorders (23).

In summary, RTW pursues the intentions of disparate groups such as employers, colleagues, unions, health professionals, as well as RTW coordinators and insurers (18). In this study, we focused on OPs as health professionals and employees as affected individuals. Given that the OPs know the workplace and also, by virtue of the pre-RTW consultations, the employees' personal situations, they can suggest appropriate interventions (e.g., change of workplace in case of tasks the employees can no longer perform due to their symptoms) (18). Through follow-up, the OPs can monitor the interventions' implementation and, if necessary, initiate further adjustments by involving the supervisors in the event of specific problems. The employees themselves function as experts with respect to their own condition and can opt for most suitable interventions. In line with the Shared Decision Making (SDM) model, in which the doctor and the patient take mutual decisions, emphasizing the patient's self-efficacy (24), this study compared both OPs and affected employees to gain a comprehensive understanding of successful RTW after depression.

Numerous studies have examined employees' RTW after a depressive episode, discussing a variety of related interventions (21, 25). An intervention is defined as any action that initiates or facilitates work and is intended to treat or rehabilitate an employee diagnosed with depression (25). Typically, interventions take into account work content, work organization, psychosocial factors, work environments that support employees suffering from depression and provide access to evidence-based care (26), helping reduce depressive symptoms, improve employees' physical and mental health and lower costs for employers by reducing sick days (27, 28). The effectiveness of individual interventions has been widely demonstrated (29). LaMontagne et al. (30) have shown that interventions at the organizational level have a more comprehensive effect on employees' mental health than those at the individual level. Organizational interventions often simultaneously benefit many employees and are usually already implemented at the beginning of the RTW, thus simultaneously ensuring prevention of exposure and disease (31). According to Bosma et al. (17), an intervention for employees with chronic conditions should focus on altering the work environment. Simultaneous initiation of multiple interventions in disparate areas of the workplace has been found to lead to greater intervention success (31). Most of the existing literature on RTW of employees after a depressive episode examines the time window before and during RTW. However, Dewa et al. (6) have found that, after completion of the RTW process, employees remain at risk of repeated sickness absence. This is due to the course of the disease, which is typically chronic with a high risk (30–70%) of early relapse (10). Given the strong evidence of an association between prolonged depression and work disability, achieving sRTW is highly relevant (32) both for employers and affected employees (27, 33). All facilitating factors and useful interventions need to be explored to help pave the way for sRTW, contributing to the creation of work environments that minimize the risk of depression relapse.

Though previous research has examined different workplace-based interventions relevant to employees' RTW after a depressive episode, this study provides an assessment of various factors facilitating or hindering RTW, as well as the relevant interventions and conditions for sRTW. As the interventions are constantly evolving, there is a need for an up-to-date analysis. To our knowledge, this is the first study that combines the practical experience of OPs, whose perspectives are scarcely evaluated, with quantitative research. Interviews with senior OPs, followed by a questionnaire survey with both OPs and employees, helped us obtain relevant empirical evidence, indicating new ways of potentially closing this research gap.

**Figure 1:**
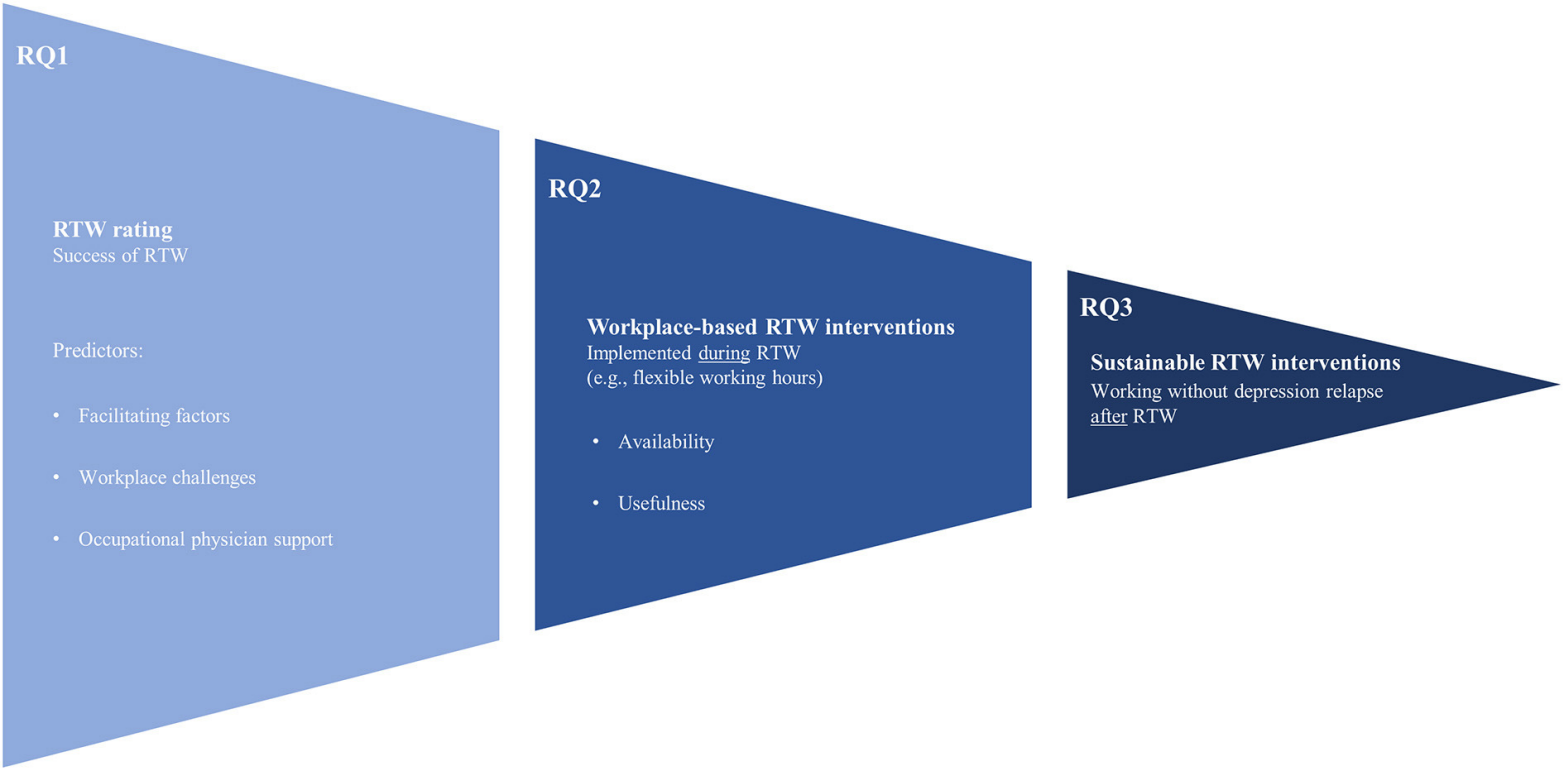
Schematic illustration of return to work (RTW) process and investigated research questions (RQ).

Our first research question (RQ1) examined how the facilitating factors, workplace challenges and OP support influence the RTW rating that indicates successful RTW, considering the perspectives of OPs (RQ1a) and affected employees (RQ1b). In research question 2, we compared OPs' and affected employees' assessments of workplace-based interventions during RTW. The availability (RQ2a) and usefulness (RQ2b) of interventions were examined for both groups. Thirdly, we explored differential assessments of interventions for prevention of depression relapse, across OPs and employees (RQ3). The assessed interventions were measured to operationalize the impact on sRTW. Please see Figure 1 to get an overview of the study.

## Methods

### Design

We applied a mixed methods approach to combine the advantages of qualitative and quantitative research, with the former having been conducted by interviewing OPs (Study 1) to identify relevant factors and interventions present in German companies for RTW success. Study 1 was followed by a questionnaire survey with OPs and employees after depressive episodes (Study 2) to statistically compare the relevance of the various factors and to identify the divergent views of the OPs and the affected employees. This sequential exploratory design (34) allowed us to transfer information and findings from Study 1 to Study 2. Both studies were approved by the ethics committee of the study center.

### Study 1

Study 1 aimed at generating suitable questionnaire items regarding successful RTW interventions after sick leave due to depression. Therefore, we developed an interview guide by modifying parts of the “success case method” (35) and items about the working conditions grouped into work patterns according to the Joint German Occupational Safety and Health Strategy (36). Following initial literature research, as outlined in the introduction, we used brainstorming techniques with three occupational health researchers from our institute and one OP from a large company to include as many relevant aspects as possible in our interview guide. Thus, an interview guide was developed. Recruitment invitation emails were sent to 40 senior OPs three times between January and May 2021. As all the invited OPs were part of the German Society for Occupational and Environmental Medicine, they were experienced in current occupational health research, and worked in large companies. Five female OPs [professional experience in years: M(SD) = 24.0 (9.41)] agreed to participate in our in-depth semi-structured expert interviews [duration M(SD) = 47.1 min (1.73 min)]. New participants were recruited and interviewed until no new relevant information could be determined, indicating sufficient data collection. Interviews were conducted via video phone calls and were digitally recorded. Prior to the interviews, all participants provided written informed consent and privacy statement for recording and further use of the interview results for research purposes. All interviews were transcribed verbatim, and data analysis according to Mayring (37) was carried out by two independent coders. A more detailed description of methods and results of Study 1 are shown in the Supplementary material. Based on the results, a questionnaire for Study 2 was developed with the topics “predictors of RTW rating,” “availability and usefulness of workplace-based RTW interventions” and “sustainable RTW interventions.”

### Study 2

#### Sample and procedure

For Study 2, data from a self-selective sample of two groups were used. Group 1 was composed of OPs who regularly deal with employees and thus accompany them in their RTW process, while Group 2 consists of employees who returned to work after a depressive episode. The online questionnaire was generated and made available using SoSci Survey (38). Participants were recruited from November 2021 through March 2022 in Germany.

OPs were recruited via the social media sites of professional societies and their exclusive mailing lists. The questionnaire was also advertised at national congresses of OPs. Out of the 405 OPs who had started the questionnaire, 211 reached the last page, which was necessary for inclusion. Many OPs (*n* = 61) dropped out on page 5, where they had to answer open questions about the companies they are consulting. We excluded 29 OPs due to missing data in at least one variable relevant for successive regression analyses (see below). Additionally, two OPs with the gender “diverse” were excluded, since gender was used as a control variable for the regression analysis. In total, 180 OPs were included in the analyses.

To recruit employees, we used online depression support groups and depression forums along with newsletters from professional societies regarding psychiatric disorders and exclusive mailing lists of expert associations. Further, 1,400 flyers and 90 posters were printed and distributed to psychiatric wards, hospital outpatient clinics, psychiatrists, psychotherapists, and pharmacies. The inclusion criteria for participation in Group 2 were being employed, aged at least 18 years, diagnosed with depressive disorder, and having started at least one RTW after a depressive episode. A total of 445 employees started the questionnaire, of which 270 reached the last page. Three entries were excluded due to missing data, five entries were excluded due to “diverse” gender, and 15 were excluded for not meeting the inclusion criteria at all. One employee with unreliable data (number of episodes = 50) was deleted. In addition, we excluded 54 employees due to missing data in at least one regression variable (see below). The number of employees included in the analyses was 192.

#### Sample characteristics

The OP sample (Group 1) consisted of 180 participants. The mean age was 52.9 years (SD 9.2y) and 106 (58.9%) of the participants were female. The employee sample (Group 2) included 192 participants, with a mean age of 45.3 years (SD 11.1y) and 136 (70.83%) females. The prevalence of current depression in employees was 49.5%. Additional descriptive parameters can be found in Table 1. A correlation table with all regression variables is shown in the Supplementary material (Supplementary Table S1).

**Table 1:** Description of study population.

	***n* (%)**	**Mean (SD)**	**Median**	**Range**
**Occupational physicians (*****N*** = **180)**
**Gender**
Male	74 (41.11)			
Female	106 (58.89)			
Age		52.88 (9.24)	55	30–75
Full-time work	131 (72.78)			
Part-time work	49 (27.22)			
Working time in hours/week		37.17 (12.02)	40	0–70
**Employment**
Employed	88 (48.89)			
External	42 (23.33)			
Inter-company	38 (21.11)			
Other	12 (6.67)			
Professional experience in years		16.86 (10.25)	16	1–40
Company size				
Micro-entities (<10 employees)	0 (0.00)			
Small companies (<50)	3 (1.67)			
Medium-sized companies (<250)	15 (8.33)			
Large companies (>250)	161 (89.44)			
More than one accompanied company	80 (44.44)	34.11^a^ (46.04^a^)	15^a^	2–200^a^
**Employees (*****N*** = **192)**
**Gender**
Male	56 (29.17)			
Female	136 (70.83)			
Age		45.27 (11.14)	47	20–71
Full-time work	108 (56.25)			
Part-time work	84 (43.75)			
Working time in hours/week		29.4 (13.49)	30	0–60
Company size				
Micro-entities (<10 employees)	17 (8.85)			
Small companies (<50)	28 (14.58)			
Medium-sized companies (<250)	38 (19.79)			
Large companies (>250)	109 (56.77)			
Sick leave due to depressive episode	192 (100.00)			
Employer knows about depression	141 (73.44)			
Number of depressive episodes		3.81 (3.29)	3	0–25
Longest sickness absence in months	188 (97.92)	10.63 (9.82)	8	1–98
Currently in RTW process	16 (8.33)			
Acute depressive syndrome (PHQ-9)	95 (49.48)			

OPs, occupational physicians, ^a^80 OPs with more than one accompanied company.

#### Measures

##### Demographic characteristics

We included age and years of professional experience of OPs and age and gender of employees.

##### RTW rating

This variable is an indicator of successful RTW. Participants (both OPs and employees) assessed the overall RTW process (“Overall, how would you rate the process of your/the employee's reintegration?”) on a five-point Likert-type item (“very bad” = 1; “very good” = 5). In this variable, answers from participants who had returned to work without accompanied RTW process (from one day to the next) as well as those from participants who had returned to work step-by-step were included. The latter refers to an accompanied, standardized, stepwise (successively increasing the number of working hours) RTW, offered to every employee in Germany with a sickness absence of more than 6 weeks. Employees can choose between the two RTW options.

##### Factors facilitating RTW

The presence of the five most important factors, according to the interview findings, that presumably facilitate employees' RTW after a depressive episode were surveyed, using a five-point Likert-type item (“disagree” = 1; “agree” = 5). Those factors related to adequate RTW coordination, team and supervisor support and prevention at the workplace (e.g., “There was further training for employees on the prevention of mental illness”). The five items were used as single variables in the regression analysis.

##### Workplace challenges

Both OPs and employees assessed three challenges for employees in the workplace (e.g., “During my return to work, I/the employees experienced stigmatization in the workplace due to my/his/her illness”), using a five-point Likert-type item (“not true” = 1; “true” = 5). We included all three items as single variables in the regression analysis.

##### OP support

Support by OPs for employees was assessed in both groups with seven items (e.g., “Was the OP involved in the RTW process?” or “Does the OP support the implementation of intervention measures?”), with answers indicating “yes” (=1) or “no” (=0). All items were added up to a sum scale. A high score indicated closer contact between OPs and employees and a higher level of support from the OP. Cronbach's alpha was 0.60 for the physicians and 0.82 for the employees.

##### Work performance

We assessed two items of employees' work performance during RTW, namely occurrence of depressive symptoms and reduced work performance, applying a five-point Likert-type item (“not true” = 1; “true” = 5).

##### Present depressive symptoms

Depressive symptoms in employees were measured through the German version of the Patient Health Questionnaire with 9 items (PHQ-9, (39)). The sum scale's internal consistency was α = 0.86. The employees were categorized in two groups, with and without depression, by using the PHQ-9 coding scheme (40).

##### Workplace-based RTW interventions

A total of 14 items were extracted from the Study 1 results to measure workplace-based RTW interventions. Following the Joint German Occupational Safety and Health Strategy (36), four categories were considered: work content and task (e.g., “I can adapt tasks to my work performance”), organization of work (e.g., “flexible working hours”), social relations (e.g., “team workshops or discussions in case of conflicts”), and working environment (e.g., “work without noise”). First, we asked both the OPs and the employees if the intervention was available in the workplace (“no” = 0; “yes” = 1; “don't know” = −9). Secondly, we assessed the perceived usefulness of each intervention in the context of an RTW after a depressive episode, applying a four-point Likert-type item (“not useful” = 1; “useful = 4”). All items were used as single variables in our analysis.

##### Sustainable RTW

Study 1 indicated several interventions and conditions to prevent the relapse of a depressive episode after completed RTW. In total, 12 items (e.g., “A relapse of a depressive episode can be prevented by a wide range of support from contact persons”) were assessed with a five-point Likert-type item (“disagree” = 1; “agree” = 5). All 12 items were analyzed as single variables in the *t*-test analysis. Long-term prevention of relapse achieves sRTW, which is why these terms are used synonymously in our study.

### Statistical analyses

All analyses were performed using R Version 2021.09.1+372 (41). To explore the predictors of RTW rating, three multiple linear regression models were fitted for each group, respectively. We structured the models hierarchically to analyze the added predictive power of each group of predictors. Model 1 included factors facilitating RTW and workplace challenges. In Model 2, OP support was added. Models 1 and 2 included the same variables for OPs and employees. In Model 3, we added age and professional experience (for OPs) or age, gender, occurrence of depressive symptoms during RTW, reduced work performance and severity of depressive symptoms (for employees) to account for relevant control variables. RTW rating was taken as a dependent variable for all three models in both groups. Effect sizes were interpreted according to Cohen (42). The 14 items of workplace-based RTW interventions were tested for significant differences between OPs and employees in terms of availability only comparing “yes” and “no” answers (excluding “do not know”) and usefulness (response scale was dichotomized). Secondly, we applied Chi-square difference tests for each item with Bonferroni's correction. To examine the differences between 12 interventions for sRTW, an unequal variance *t*-test (43) with Bonferroni's correction was performed.

## Results

### Predictors of RTW rating

RQ1 sought to determine which interventions within the RTW process have the greatest impact on RTW rating, for both OPs (RQ1a) and employees (RQ1b) after a depressive episode. The results of the multiple regression analyses for OPs and employees are shown in Table 2. In Model 3 (OPs), “employee training on mental illness prevention” and “support by the occupational physician” were positive predictors of RTW rating. A negative impact on RTW rating is predicted by “stigmatization in the workplace,” “working under conditions in the workplace that promote depressive symptoms” and “no support by colleagues.” In contrast to Models 1 and 2, “adequate coordination between the employee, supervisor, works council, occupational physician” and “understanding and appreciation in conversations” lacked significance when control variables were considered. The adjusted variance explained (adj. R^2^ = 0.47) improved marginally by adding more predictors between Models 1 and 3. All in all, RQ1a can be addressed as “employee training on mental illness prevention” having the most positive impact on the outcome, while “stigmatization in the workplace” having the most negative impact.

**Table 2:** Standardized regression coefficients for RTW rating separated by OPs and employees.

	**Dependent variable: RTW rating**					
	**OPs (*****N*** = **180)**			**Employees (*****N*** = **192)**		
	**Model 1**	**Model 2**	**Model 3**	**Model 1**	**Model 2**	**Model 3**
**Predictors**						
Adequate coordination	0.18^*^	0.13	0.13	0.13^*^	0.12^*^	0.13^*^
Understanding and appreciation in conversations	0.15^*^	0.15^*^	0.14	0.21^**^	0.21^**^	0.21^**^
Support by supervisor	0.11	0.10	0.10	0.10	0.10	0.08
Additional employee for the team during RTW	−0.03	−0.02	−0.02	0.09	0.08	0.08
Employee training on mental illness prevention	0.21^**^	0.18^**^	0.18^**^	0.12^*^	0.10	0.10
Stigmatization	−0.15^*^	−0.17^*^	−0.16^*^	−0.20^**^	−0.20^**^	−0.21^**^
Workplace conditions promoting depression	−0.19^**^	−0.15^*^	−0.15^*^	−0.14^*^	−0.14^*^	−0.12^*^
No support by colleagues	−0.14^*^	−0.13^*^	−0.13^*^	−0.16^*^	−0.16^*^	−0.17^**^
Support by OP		0.18^**^	0.16^*^		0.06	0.05
**Control variables OPs**
Age			−0.04			
Years of professional experience			0.10			
**Control variables employees**
Age						0.10^*^
Gender						0.07
Symptoms during return to work						−0.06
Reduced work performance during RTW						0.04
Current depressive symptoms						0.01
Constant	3.21^**^	2.85^**^	2.91^**^	2.65^**^	2.63^**^	2.18^**^
Adjusted R^2^	0.451	0.474	0.472	0.509	0.510	0.518
Δ Adjusted R^2^		0.023	0.002		0.001	0.008

OP, occupational physician; RTW, return to work; R^2^, explained variance;

* p < 0.05;

** p < 0.01.

Model 3 (employees) showed the same negative predictors as Model 3 (OPs) and demonstrated the positive impact of “adequate coordination between the employee, supervisor, works council, occupational physician” and “understanding and appreciation in conversations.” The adj. R^2^ (0.52) improved slightly between the models. RQ1b can be answered with “understanding and appreciation in conversations” having the most positive and “stigmatization in the workplace” having the most negative effects. Although an inferential statistical comparison of non-nested models is not valid, our results demonstrated that OPs and employees weighted the predictors of successful RTW process differently.

### Availability and usefulness of workplace-based RTW interventions

**Figure 2:**
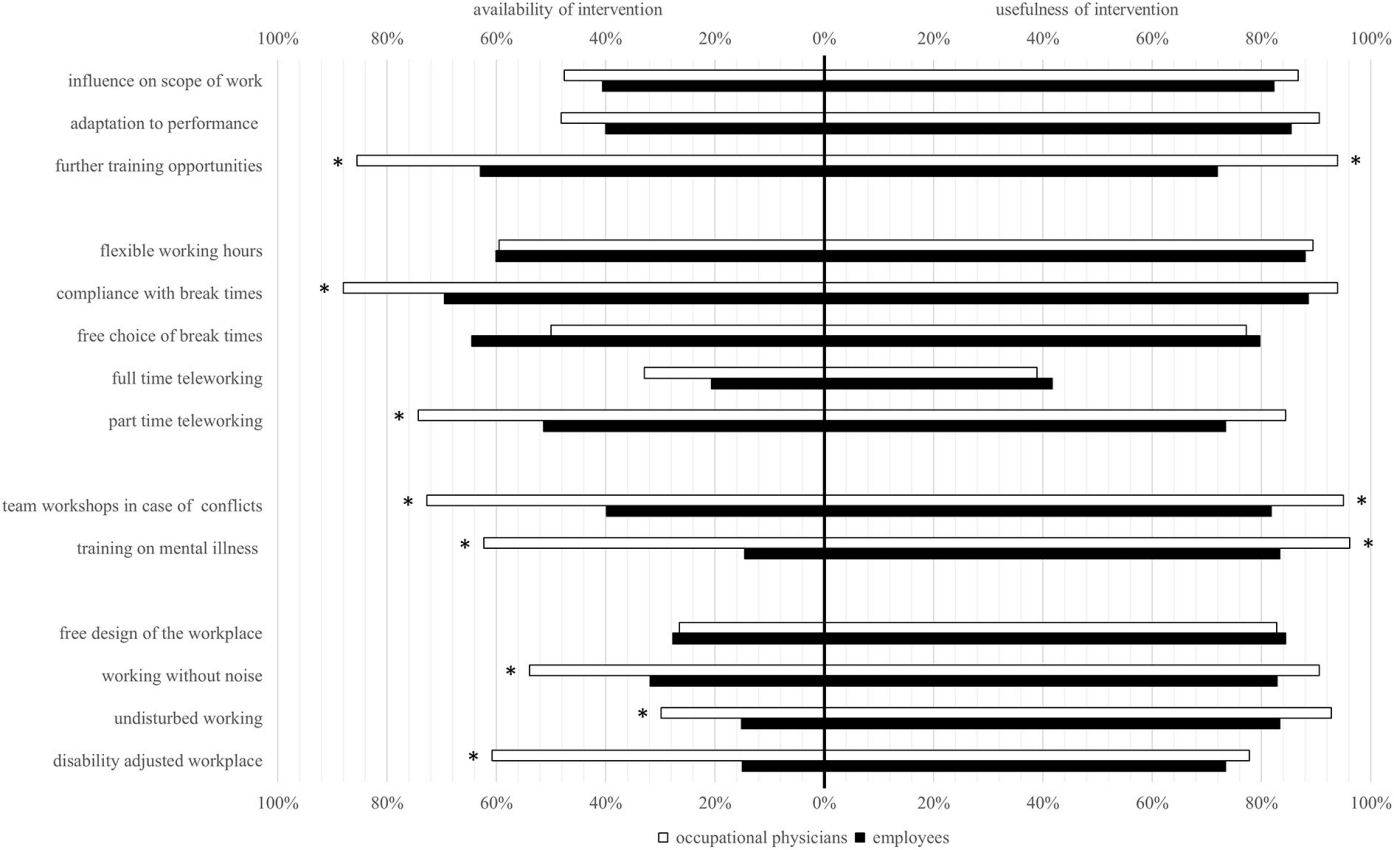
Availability and usefulness of workplace-based interventions.

Figure 2 compared perceptions of the availability (RQ2a) and usefulness (RQ2b) of workplace-based RTW interventions across OPs and employees. Addressing RQ2a, in eight out of 14 considered workplace-based interventions, OPs reported a higher availability than employees (*p* < 0.005), most noticeably in “training on mental illness,” “disability adjusted workplace,” and “team workshops in case of conflicts.” Considering the usefulness of some interventions in RQ2b, “further training opportunities,” “training on mental illness,” and “team workshops in case of conflicts” were rated higher by OPs than employees (*p* < 0.005).

### Sustainable RTW interventions

RQ3 examined differential assessments of OPs and employees on whether or not interventions are suitable in preventing relapse (see Table 3). Employees rated “sufficient time before RTW” (*t* = −3.13; *p* < 0.02; Δ mean = 0.32) and “better financial compensation for the employee before returning to full-time employment” (*t* = −6.00; *p* < 0.01; Δ mean = 0.76) as more suitable, while a “wide range of support from contact persons” (*t* = 3.02; *p* < 0.03; Δ mean = 0.31) was considered more appropriate by OPs.

**Table 3:** Relevance of interventions fostering sustainable RTW assessed by OPs and employees.

**Item**	**Mean (SD) OPs**	**Mean (SD) employees**	**Δ Mean**	***t*-Value**	***p*-Value**
Supervisor support	4.39 (0.77)	4.52 (0.81)	0.13	−1.48	0.139
Resolving conflicts with supervisor	4.38 (0.74)	4.27 (0.95)	0.11	1.28	0.203
Team support	4.30 (0.86)	4.36 (0.87)	0.06	−0.66	0.509
Support from contact persons	4.23 (0.82)	3.92 (1.15)	0.31	3.02	0.003^*^
Resolving conflicts with team	4.15 (0.93)	4.03 (1.00)	0.12	1.24	0.218
Better information about support from contact persons	4.04 (0.93)	3.90 (1.11)	0.14	1.30	0.194
Fixed task field	4.03 (0.93)	4.15 (1.05)	0.12	−1.10	0.273
Sufficient time before RTW	3.92 (1.00)	4.24 (1.02)	0.32	−3.13	0.002^*^
Personal coaching	3.85 (0.90)	3.95 (1.12)	0.10	−0.98	0.326
Permanent supervisor	3.80 (1.00)	3.90 (1.18)	0.10	−0.85	0.397
Better financial compensation before and during RTW	3.05 (1.15)	3.81 (1.29)	0.76	−6.00	0.001^*^
Workplace transfer	2.94 (0.67)	2.93 (1.35)	0.01	0.16	0.874

Unequal variance t-test, alternative hypothesis two-sided (OPs N = 180; employees N = 192); RTW, return to work; OPs, occupational physicians;

* p < 0.0042 (level of significance after Bonferroni's correction), ordered by Mean OPs, all variables ranged from 1 to 5.

## Discussion

This mixed methods study sought to investigate possible approaches for sustainable RTW (sRTW) after a depressive episode, identifying factors that optimize the RTW. To that end, we brought to light the perspectives of both OPs, as experts, and affected employees, identifying priorities and divergences. We examined the predictors of RTW rating and assessed the availability and usefulness of workplace-based RTW interventions and suitable interventions for sRTW.

### Predictors of RTW rating

We examined the predictors of OPs' and employees' overall RTW. The finding that, for both groups, stigmatization was the strongest negative predictor of overall RTW rating is in line with previous research, which found stigmatization to harmfully impact the cooperation between employees with chronic health conditions, such as depression, and their colleagues or supervisors (44). Nielsen et al. (45) have argued that stigma attached to mental health problems may even lead to dismissal when the employer anticipates low productivity after RTW. For employees, especially anticipated stigmatization can interfere with the RTW process (46).

Employee prevention training on mental health was found to be the most important positive predictor for OPs. However, LaMontagne et al. (47) have shown that the implementation of a job stress intervention and mental health training program lead to short-term improvements in employees' health literacy. Another meta-analysis has found psychoeducation to be truly effective in reducing depressive symptoms (48), thus improving RTW (21), and mental health literacy, rather than physical activity or vitamin supplementation, to be the only suitable preventive intervention vis-a-vis depressive symptoms within a 6-week period (49). Future research, therefore, should explore ways to adapt health literacy programs to ensure longer-lasting benefits.

Typically, with many years of experience in their organizations' RTW processes, OPs are more familiar than most employees with interventions such as employee prevention training on mental health. Therefore, they monitor therapeutic and organizational interventions (18). Moreover, OPs are often involved in the development and implementation of preventive activities (18). Thus, compared to the employees, the OPs' better understanding of these interventions and, in some cases, personal involvement may lead them to evaluate these interventions as more facilitating for RTW. Our observation of the employees reporting limited availability of interventions (RQ2) corroborates this notion, suggesting that these interventions may be deemed less beneficial owing to either a lack of their use or awareness about them.

We found the employees to rate interpersonal understanding and appreciation highest for successful RTW, while the lack of collegial support was a hindering factor in both groups. Previous research has clearly shown that social support is essential for successful RTW, and that it is more relevant to mental than physical disorders (19, 21, 22). In their meta-analysis, Ervasti et al. (21) have illustrated how the lack of social support is linked to slower return from depression-related absence from work, while high social support facilitates quicker RTW. Therefore, interventions focusing on awareness and communication skills are crucially important and should be further developed.

In our study, both groups considered adequate coordination between the different stakeholders at the workplace as a facilitating factor for RTW, corroborating the conclusion of de Vries et al. (19) that adequate coordination and guidance are key to the RTW process and that a new type of intervention, therefore, is needed. Their qualitative study has found that employees emphasize emotional support while OPs prioritize adapted work environments, indicating the importance of addressing both perspectives.

### Availability and usefulness of workplace-based RTW interventions

We investigated the availability and usefulness of RTW interventions, evaluated by both groups. OPs generally reported more availability of interventions for employees, and rated them as more useful than their counterparts did, although the assessed usefulness was generally high in both groups. We noticed a lack of knowledge about the availability of workplace-based interventions, particularly among employees (7.14% answered “don't know” when asked if an intervention is available in their company; data not shown). Naturally, as experts in their organizations who regularly deal with RTW of employees suffering from depression, OPs have a broader knowledge of existing RTW interventions. To reduce the gap between the awareness of available interventions and their application, companies and OPs should pay special attention to fully informing their employees and involving them in RTW processes, proactively raising awareness of prevention services. In terms of usefulness, OPs rated employee trainings and team workshops more important than the employees did. While these interventions may have proved useful to most employees, those still struggling with acute depressive symptoms (e.g., lack of energy and confidence, insomnia or inability to concentrate) (50) may benefit more from interventions addressing the direct working environment (e.g., free design of the workplace, working without noise), requiring less effort and active participation from the employee. Establishing organizational interventions may require greater financial effort and resources on the part of the employer, so it is conceivable that the employer would rather encourage the employee to participate in individual interventions. However, a systematic review of work-focused interventions has shown that organizational RTW interventions are cost-effective for both the employer and society (33). Therefore, it is likely to be beneficial for employers to prioritize organizational interventions, given that they can have a more comprehensive impact compared to interventions at an individual level (30).

There is not one ideal intervention for the employees' RTW after a depressive episode, but the wide spectrum of simultaneously offered interventions seems promising (31). Depending on individual and contextual factors, some employees may benefit from trainings, workshops, and emotional support, while others may require adapted working conditions for a sustainable RTW process.

### Sustainable RTW interventions

We sought to explore the existing approaches to sRTW interventions and identify new avenues, shedding light on divergent views of OPs and affected employees. We found that, compared to OPs, employees considered better financial compensation before returning to full-time employment and sufficient absence time before restarting full-time work as more important. It is conceivable that the lack of employees' financial reserves may lead to early RTW by employees still suffering from acute depressive symptoms, which may complicate an sRTW. Grahn et al. (51) have found that employees who have a longer rehabilitation period are more likely to achieve RTW, with those who are sole earners, and are fully responsible for their families' financial security, showing a less favorable course of depressive symptoms (52). As employees have to deal with the consequences of their sickness absence on a daily basis, it is possible that they, compared to OPs, prioritize both the financial aspects and sufficient time. After all, better financial compensation before returning to full-time employment can help reduce income-related stress and prevent premature RTWs. Thus, a combination of better financial compensation and sufficient absence time before RTW can promote a successful sRTW. From the standpoint of OPs, however, who prioritize the adaption of work environments (19), the picture is significantly different, which has already been shown in other studies. We found that, compared to the employees, the OPs considered a “wide range of support from contact persons (e.g., works council, human resource management, physicians, social counseling, confidential counselors)” more important for both successful RTW rating (RQ1) and sRTW (RQ3). In contrast, de Vries et al. (19) had found that items from the supportive health care cluster (support from professionals involved in RTW facilitation, namely psychologists and general physicians) were equally important for employees and OPs. This discrepancy may be due to the fact that physicians were directly addressed in our questionnaire as they had been listed as contact persons. Floer et al. (24) have observed that physicians assess their own behavior during the conversation with the patient more positively compared to how patients remember the physician's behavior. Overall, it is noticeable that both groups rate the same interventions as most suitable (highest mean) for relapse prevention, resolving conflicts with the supervisor as well as supervisor and team support. Good interpersonal relationships seem to be a precondition for functioning collaborations in the daily work routine after RTW, and thus immensely important for sRTW (9).

### Implications for future research and RTW practice

This study was based on the practical experience of OPs (expert interviews) followed by a questionnaire covering the perspectives of both OPs and affected employees. Our results suggest that good cooperation and coordination between the OP and the employee is crucial for the sustainable success of RTW, given that different priorities exist in the RTW process. Therefore, Shared Decision Making (SDM) can play a key role in planning the RTW primarily in the employee's interest and based on the expert knowledge of the OP. SDM can effectively facilitate understanding and appreciation in conversations, which are essential components for a successful RTW, particularly for employees. Future research should investigate how joint actions between OPs, employees and other shareholders can be facilitated, reconciling possible conflicts of interest (e.g., rapid productivity recovery vs. sustainable rehabilitation). To ensure sRTW, research should also focus on possible ways of reducing the financial anxiety and hardship of employees with depression. As the RTW process imposes costs on the employer (53), it employers would be in the interest of employers to reintegrate employees as sustainably as possible to avoid further expenses due to sickness absence and repeated RTWs. As many OPs mentioned, a wide range of support from contact persons, especially low-threshold intervention, is needed. At the end of the questionnaire, 45 (25.0%) OPs and 45 (23.4%) employees, provided free text entries, often expressing a desire to improve the RTW process. This feedback further underlines the relevance of more multi-perspective and interdisciplinary research on RTW for employees after a depressive episode.

### Strengths and limitations

To the best of our knowledge, this is the first quantitative study comparing the perspectives of OPs and employees with respect to workplace-based RTW interventions after a depressive episode.

OPs and employees from various companies were interviewed nationwide to obtain cross-sectional data of workplace and RTW conditions. However, this also implies that surveyed OPs and employees came from different companies, representing different work contexts, limiting one-to-one comparisons of different groups of participants.

We only surveyed interventions related to the workplace, as it is an important part of most employees' (daily) life, with workplace adjustments having the potential to reach many people at once and play a key role in the success of RTW. Although many interventions have been subject to research in various studies, combined and simultaneous assessment of interventions, as done in this study, was so far missing.

On the other hand, Cronbach's alpha for the OP support sum scale (0.60) was relatively low. Low Cronbach's alpha is often observed for short scales of 10 or fewer items that are more heterogeneous and therefore decrease inflation of internal consistency. Because the OPs support sum scale was only applied for group statistics, we followed the common practice of emphasizing measurement efficiency over internal consistency (54).

Also, given that it was a cross-sectional study, it was not possible to draw causal conclusions and a recall bias cannot be ruled out. Future research should use a longitudinal design, with measurements before, during and after RTW for the effectiveness of an intervention.

The period of data collection might have been influenced by various factors: The COVID-19 pandemic and partial lockdowns made recruitment via posters and flyers in suitable places (e.g., psychotherapy offices) less effective. The simultaneous engagement of OPs in the vaccination campaign in Germany and the recruitment in the pre-Christmas period led to lower initial responses. Although a total of 445 employees had started the questionnaire, only 270 completed it. The length of the questionnaire (15–20 min) coupled with the fact that people suffering from acute depression lack the ability to concentrate (50) may explain the loss of participants. The participants who dropped out showed no substantial differences in study variables compared to participants who responded until the end. Despite a rather small sample size due to these reasons and the strict inclusion criteria, the power (=1) of all models in the regression proved to be sufficient.

Some OPs work as external or inter-company OPs, which means they work for more than one company. In the questionnaire, they were asked to choose a company that best matched the issue. Therefore, all other companies were not represented. Presumably, the larger company performing more RTWs and offering more workplace-based interventions was preferred by those OPs. It may also be advantageous for OPs to be familiar with several places of work, as this gives them a broader range of experience that is less dependent on contextual or situational factors.

The employees' answers on RTW rating might have been influenced by potential acute depressive symptoms and moods, resulting in poor evaluations of past events (55). However, the evaluation of the PHQ-9 indicated that about half of the employees did not suffer from depression at the time of participation. In addition, current depressive symptoms were controlled for in the regression analysis.

## Conclusions

Our study reveals how priorities in the RTW process differ between OPs and employees after a depressive episode, identifying themes for future research about post-depression RTW. Future studies should apply a longitudinal design to determine causality and consider a multi-perspective and interdisciplinary approach. In addition, a follow-up from the beginning of RTW to the period after RTW would be needed to avoid recall bias. The comparison between OPs and employees indicates that a basis of trust, regular communication, financial support and the consideration of both perspectives is essential to ensure successful and sustainable RTW.

## Data availability statement

The datasets presented in this article are not readily available because of data privacy issues. Requests to access the datasets should be directed to richard.ersel@rwth-aachen.de.

## Ethics statement

The study was approved by the Local Ethics Committee of the Medical Faculty of RWTH Aachen University (EK 025-21). The patients/participants provided their written informed consent to participate in this study.

## Author contributions

RE, PG, and JL were responsible for the conception and study design and developed the questionnaire. RE and JL contributed to data acquisition. RE and RP conducted the analyses. RE created the figures and tables and the first draft of the manuscript. All authors gave substantial intellectual input during the preparation process, contributed to the interpretation of this dataset, commented on previous versions of the manuscript, and read and approved the final manuscript.

## Conflict of interest

The authors declare that the research was conducted in the absence of any commercial or financial relationships that could be construed as a potential conflict of interest.

## Publisher's note

All claims expressed in this article are solely those of the authors and do not necessarily represent those of their affiliated organizations, or those of the publisher, the editors and the reviewers. Any product that may be evaluated in this article, or claim that may be made by its manufacturer, is not guaranteed or endorsed by the publisher.
