# Supplementary material for: Sustainable return to work after depression - A comparative study among occupational physicians and affected employees
Source: Front Public Health. 2022 Oct 6;10:946396. doi: 10.3389/fpubh.2022.946396 (PMC9583521; doi:10.3389/fpubh.2022.946396)
Supplement: Supplementary file 1 [file Data_Sheet_1.docx]

Supplementary Material

# Correlation tables

**Table S1a.** Means, standard deviations, and Pearson correlations for occupational physicians.

| Variable | *M* | *SD* | 1 | 2 | 3 | 4 | 5 | 6 | 7 | 8 | 9 | 10 | 11 |
| --- | --- | --- | --- | --- | --- | --- | --- | --- | --- | --- | --- | --- | --- |
| 1. RTW rating | 3.53 | 0.77 |  |  |  |  |  |  |  |  |  |  |  |
| 2. adequate coordination | 3.46 | 1.15 | .43** |  |  |  |  |  |  |  |  |  |  |
| 3. understanding and appreciation in conversations | 3.89 | 0.99 | .53** | .54** |  |  |  |  |  |  |  |  |  |
| 4. support by supervisor | 4.22 | 1.00 | .42** | .44** | .45** |  |  |  |  |  |  |  |  |
| 5. additional employee for the team during RTW | 1.87 | 0.99 | .13 | .22** | .12 | .15* |  |  |  |  |  |  |  |
| 6. employee training on mental illness prevention | 3.18 | 1.44 | .38** | .32** | .35** | .26** | .20** |  |  |  |  |  |  |
| 7. stigmatization | 2.62 | 0.87 | -.41** | -.07 | -.34** | -.17* | -.06 | -.12 |  |  |  |  |  |
| 8. workplace conditions promoting depression | 3.08 | 0.80 | -.42** | -.20** | -.29** | -.23** | -.11 | -.02 | .48** |  |  |  |  |
| 9. no support by colleagues | 2.42 | 0.81 | -.40** | -.05 | -.37** | -.32** | -.08 | -.08 | .47** | .39** |  |  |  |
| 10. support by OP | 5.35 | 1.49 | .40** | .40** | .27** | .26** | .08 | .29** | -.04 | -.23** | -.09 |  |  |
| 11. age | 52.88 | 9.24 | .09 | .01 | .17* | .01 | -.13 | -.22** | -.03 | -.17* | -.11 | .11 |  |
| 12. years of professional experience | 16.86 | 10.25 | .18* | .06 | .19* | .05 | -.10 | -.10 | -.12 | -.16* | -.11 | .18* | .80** |

Notes: M and SD are used to represent mean and standard deviation, respectively. * indicates p < .05. ** indicates p < .01.

**Table S1b.** Means, standard deviations, and Pearson correlations for employees.

| Variable | *M* | *SD* | 1 | 2 | 3 | 4 | 5 | 6 | 7 | 8 | 9 | 10 | 11 | 12 | 13 | 14 |
| --- | --- | --- | --- | --- | --- | --- | --- | --- | --- | --- | --- | --- | --- | --- | --- | --- |
| 1. RTW rating | 2.91 | 1.15 |  |  |  |  |  |  |  |  |  |  |  |  |  |  |
| 2. adequate coordination | 2.75 | 1.39 | .43** |  |  |  |  |  |  |  |  |  |  |  |  |  |
| 3. understanding and appreciation in conversations | 3.41 | 1.35 | .59** | .46** |  |  |  |  |  |  |  |  |  |  |  |  |
| 4. support by supervisor | 3.24 | 1.49 | .44** | .47** | .49** |  |  |  |  |  |  |  |  |  |  |  |
| 5. additional employee for the team during RTW | 1.47 | 1.02 | .24** | .21** | .13 | .12 |  |  |  |  |  |  |  |  |  |  |
| 6. employee training on mental illness prevention | 1.79 | 1.26 | .32** | .26** | .25** | .28** | .26** |  |  |  |  |  |  |  |  |  |
| 7. stigmatization | 2.69 | 1.45 | -.54** | -.23** | -.60** | -.25** | -.13 | -.14 |  |  |  |  |  |  |  |  |
| 8. workplace conditions promoting depression | 3.55 | 1.35 | -.42** | -.17* | -.30** | -.19** | -.14 | -.19** | .37** |  |  |  |  |  |  |  |
| 9. no support by colleagues | 2.69 | 1.32 | -.48** | -.22** | -.41** | -.30** | -.06 | -.05 | .52** | .39** |  |  |  |  |  |  |
| 10. support by OP | 1.91 | 2.04 | .23** | .20** | .13 | .19** | .22** | .35** | -.05 | -.16* | -.05 |  |  |  |  |  |
| 11. age | 45.27 | 11.14 | .12 | -.01 | -.01 | .09 | -.03 | .06 | .05 | -.05 | .04 | .07 |  |  |  |  |
| 12. gender | - | - | .12 | .01 | .10 | .07 | .07 | .03 | -.04 | .03 | -.02 | .10 | .06 |  |  |  |
| 13. symptoms during return to work | 4.03 | 1.06 | -.19** | -.08 | -.05 | .04 | -.17* | -.10 | .12 | .36** | .10 | -.15* | -.11 | .04 |  |  |
| 14. reduced work performance during RTW | 4.20 | 0.99 | -.14 | -.09 | -.14 | .09 | -.23** | -.09 | .20** | .27** | .10 | -.13 | .10 | -.02 | .50** |  |
| 15.current depressive symptoms | 11.82 | 5.99 | -.23** | -.10 | -.15* | -.13 | -.09 | -.04 | .30** | .37** | .31** | -.12 | -.03 | .11 | .33** | .16* |

Notes: M and SD are used to represent mean and standard deviation, respectively. * indicates p < .05. ** indicates p < .01.

# Study 1

## Methods

We developed the interview guide by modifying parts of the “success case method“ (1) and included questions about workplace-based return to work (RTW) interventions and sustainable RTW (sRTW). Workplace-based RTW interventions were structured in four sections: "Work content and task", "Organization of work", "Social relationships (colleagues and supervisors)" and "Work environment", according to the Joint German Occupational Safety and Health Strategy (2). We conducted literature research as outlined in the introduction (Study 2) and used brainstorming techniques with occupational physicians (OPs) to include the most relevant aspects in our interview guide. After finalization, the interview guide was tested and reviewed in terms of content, language, and practicability by an OP in a senior position at a large German company.

Recruitment for Study 1 targeted experts on the topic of RTW after a depressive episode. We focused on senior OPs of large companies throughout Germany with a lot of experience in RTW procedures. All OPs were part of the German Society for Occupational and Environmental Medicine and therefore experienced in current occupational health research. OPs not providing care to employees with depression were excluded. Recruitment invitation emails were sent to 40 senior OPs on three dates between January and May 2021. Five female OPs [professional experience in years: M(SD) = 24.0 (9.41)] participated in our in-depth semi-structured expert interviews [duration M(SD) = 47.1 min (1.73 min)]. The interviews were conducted via video phone calls and were digitally recorded. Before the interviews, written informed consent and privacy statement were obtained from all participants for recording and use of the interview results for research purposes. New participants were recruited and interviewed until no new relevant information could be elicited, indicating sufficient data collection. All interviews were transcribed verbatim and data analysis according to Mayring (3) was carried out by two independent coders. The questions of the semi-structured interview already provided deductive categories. During analysis, additional inductive categories were created from participants' answers. Subsequently, phrases were generalized and categories with the same content were combined into main categories. A review of the category system took place at regular intervals. To increase the quality of the categories, definitions, anchor phrases and, in some cases, coding rules were documented. The anchor phrases from the interviews were assigned to the categories and sorted by the participants to allow comparisons between participants. Answers were used to generate items for the online questionnaire in Study 2. The study was approved by the local ethics committee of the Medical Faculty of RWTH Aachen University (EK 025-21).

## Results

We have summarized the results from Study 1 below. The following is not an exhaustive list of all responses but shows most common interview answers, structured according to the system of categories derived from qualitative content analysis. The various participants are abbreviated with IV and a random number to ensure anonymity.

### Facilitating factors for return to work

The interview participants reported interventions that can foster the success of RTW. These included involving supervisors by informing them about the availability of the company's interventions and "direct exchange with employees and supervisors" (IV19) in order to provide individually tailored support for employees during RTW. Furthermore, understanding and appreciation in conversations played a major role given that “a person suffering from depression is to be treated differently than someone with […] cancer or with a stroke" (IV13). Adequate coordination between the employee, supervisor, works council, and occupational physician can help to make RTW more effective. Therefore, " joint interdisciplinary cooperation [among stakeholders] for the employees" remains important (IV15).

### Obstacles and challenges

Various obstacles and challenges in the implementation of RTW interventions were mentioned. The lack of support from supervisors or colleagues can hinder the acceptance of interventions in everyday working life. "That there are also supervisors who are resistant to consultation and who think, with their text-books in their heads, that they can have every employee back the way he or she was originally." (IV19). "So, the be-all and end-all of reintegration of people with depression is really that there is understanding for each other, that both sides have understanding for each other. It's not a one-way street." (IV11). Workplaces that aggravate depressive symptoms, such as those with a "stressful environment" (IV 19), should be avoided. “Workplace disputes and poor work climate is also something we struggle with a lot in individual units.” (IV19). "Often it is also work-related illnesses, or the work has a large part in the mental illness, in the depression. And it is of course a frightening situation, the return to work" (IV17). All interview participants mentioned lower work performance after the depressive episode. As a result, employees are usually unable to do the same work as before and one needs the "capacity of colleagues to retrain him, to re-skill him, to re-integrate him" (IV15). Further, employees may struggle with illness characteristics of depression during RTW: "I may still have concentration problems, I may have an ego strength problem, that is manifold. I may still have symptoms, sleep problems and can't be on time, whatever, I have medication side effects, that is endless" (IV15). Stigmatization at work also remains a major issue: "So I think first of all, most still have to overcome prejudices, so the supervisor and also the team have to pay attention that no situations of discrimination occur, because that unfortunately still happens with mental illness in society" (IV17).

### Work content and task

In the context of "work content", the adaptation of tasks to the employee’s performance was mentioned. By "relieving the employee of certain tasks that he or she cannot perform at the moment" (IV 11) and "excluding parts of the activity that are perhaps not so conducive at the moment" (IV19), overexertion of the employee can be minimized. It is also important that employees "have a wide scope of work where they can make a difference themselves" (IV13), so that individual adaptation of the workplace can be achieved. In addition, further training and retraining are considered helpful. “That you also have the opportunity to get out of such a very clocked working world […], that you can get out of it and retrain in another area, and perhaps go into a training department […]" (IV13).

### Organization of work

In this section, the interview participants named the compliance and planning of breaks as supportive workplace-based interventions, since employees often "feel forced to forego their breaks, simply because they have so much work due" (IV11), while sometimes even "increased recovery times [...] are needed". Furthermore, almost all participants mentioned flexible working hours as an important intervention so that the employee can plan appointments relevant to their recovery: "When IRENA programs [author's note: Intensified rehabilitation aftercare] are running or there is still outpatient aftercare or therapist appointments, we already try to coordinate with the organizational units, so that the employees can [...] attend these appointments" (IV19). Furthermore, part or full-time telework was brought up as an intervention because "someone with a rather stigmatizing illness can certainly go to therapy more unnoticed during telework than if they were on site" (IV17). All interview participants agreed that part-time telework is preferable to full-time telework because personal team communication and a regular daily structure are easier to maintain in the office. "I think it is much more beneficial if telework does not account for one hundred percent of your work, but that you spend some days at home and then other days at your workplace" (IV11).

### Social relations

Team workshops and discussions in case of conflicts were mentioned as interventions to create a healthier working environment. "We have team meetings, especially when there are conflicts, and together we think about how we can better cooperate in the future" (IV11). "If we notice that employees return to conflict-ridden parts of the company, it makes a lot of sense to intervene in advance to minimize the frequency of conflicts, to really intervene in order to design a stable working environment " (IV19). Many of the interview participants recommended "training courses for supervisors on mental illness to improve understanding" (IV11).

### Working environment

The experts valued quiet working environments with appropriate noise levels and reduced interruptions (e.g., by colleagues’ requests). Likewise, sharing the office with few colleagues is to be preferred to large open-plan offices: "In principle, open offices are of course something employees with mental illnesses tend to find unfavorable" (IV17). Therefore, a free workplace design could be a solution.

## Prevention relapse

Conflicts with supervisors or colleagues were referred to as risk factors for relapse into a depressive episode, enhancing "psychological stress [...] in the long run" (IV15). If employees do not receive sufficient financial support, they risk premature RTW: "There is often a lot of economic pressure to get back into the regular job as quickly as possible" (IV19). Most of the interview participants stated that a sufficiently long and realistic timeline is key to avoiding relapses after RTW. Each employee requires a different amount of time for successful RTW, which should not be shortened by external expectations, e.g., from colleagues. For employees, training their own awareness of personal needs at the workplace is important. It is difficult to ensure someone is around all the time to take care of the employees’ well-being. Another way to prevent relapse is to offer support options and inform the employees about them in order for the employees to take advantage of them. For this purpose, the important contact points may include "works council, personnel department, medical department, social counseling, confidential employees, and immediate colleagues with whom they can exchange information" (IV11).

## Conclusion and Discussion

To provide the best possible support for RTW after a depressive episode and to prevent depression relapse, interventions from all areas of organizational prevention are required. According to the experts, these include a large scope of work for employees, flexible working hours, team workshops and conversations in the event of conflicts, and a quiet, undisturbed working environment. Particularly for relapse prevention, individually adjusted and sufficient time before RTW without financial pressure is a key criterion.

After five interviews, we found that only new company-specific factors (e.g., time difference for international team meetings as workplace challenge) could be identified through further interviews. Given the consistency with respect to the cross-company factors (e.g., flexible working hours as a workplace-based intervention) after these five interviews, we decided to focus on these most important and common factors and developed a questionnaire from the interview results to obtain quantitative results from a larger sample size.

3 **References**

1. Brinkerhoff R. The Success Case Method: Find Out Quickly What's Working and What's Not. San Francisco: Berrett-Koehler Publishers (2003).

2. Joint German Occupational Safety and Health Strategy. Recommendations for implementing psychosocial risk assessment. Berlin (2014).

3. Mayring P. “Qualitative content analysis,”. In: Mey G, Mruck K, editors. Handbook Qualitative Research in Psychology: Volume 2: Designs and processes. Wiesbaden: Springer Fachmedien Wiesbaden GmbH (2020). p. 495–511.

# Interview Guide (translated from German)

| **Section** | **Questions** |
| --- | --- |
| **Introduction** | How does the reintegration of an employee after a depressive episode take place in your company? |
|  | What is your role in supporting the employee in his/her reintegration? |
| **Facilitating factors for return to work** | What works best to facilitate the return to work of an employee after a depressive episode? |
|  | What contributes to the success of interventions for an employee's return to work after a depressive episode? |
| **Obstacles and challenges** | What challenges do companies face in the workplace in reintegrating employees after a depressive episode? |
|  | What challenges do employees face in the workplace when they return to work after a depressive episode? |
|  | What are the potential obstacles to implementing interventions for the return to work after a depressive episode? |
| **Workplace-based RTW interventions** | What interventions regarding work content and task does the company offer to support employees after a depressive episode? |
|  | What interventions regarding organization of work does the company offer to support employees after a depressive episode? |
|  | What interventions regarding social relations (e.g., supervisor or colleagues) does the company offer to support employees after a depressive episode? |
|  | What interventions regarding working environment does the company offer to support employees after a depressive episode? |
| **Prevention of relapse** | What are the main workplace-related reasons for a depression relapse? |
|  | In your experience, what do employees need to help prevent a relapse? |
